# Action towards Universal Health Coverage and Social Protection for Tuberculosis Care and Prevention: Workshop on the End TB Strategy Pillar 2 in the Western Pacific Region 2017

**DOI:** 10.3390/tropicalmed4010003

**Published:** 2018-12-24

**Authors:** Kyung Hyun Oh, Kalpeshsinh Rahevar, Nobuyuki Nishikiori, Kerri Viney, Hongjo Choi, Olivia Biermann, Hee Jin Kim, Chanly Nou, Samoeun Kim, Ge Zhu, Hui Zhang, Caihong Xu, Soth Bounmala, Maytry Senchanthixay, Enkhtamir Purevdorj, Ulzii-orshikh Khaltar, Ronald Paguirigan, Hansel Amoguis, Vu Xuan Phu, Dang Xuan Khang

**Affiliations:** 1 Korean Institute of Tuberculosis, Cheongju-si, Chungcheongbuk-do 28158, Korea; h.choi.kit@gmail.com (H.C.); hatchingbird@yahoo.co.kr (H.J.K.); 2 World Health Organization Regional Office for the Western Pacific, 1000 Manila, Philippines; rahevark@who.int; 3 World Health Organization, Avenue Appia 20, 1202 Geneva, Switzerland; nishikiorin@who.int; 4 Karolinska Institutet, 171 77 Stockholm, Sweden; kerri.viney@ki.se (K.V.); olivia.biermann@ki.se (O.B.); 5 Research School of Population Health, National Centre of Epidemiology and Population Health, Department of Global Health, Australian National University, Canberra 2601, Australia; 6 National Center for Tuberculosis and Leprosy Control, Street 278/95, Sangkat Boeung Kebg Kang II, Khan Chamkar Morn, 1217 Phnom Penh, Cambodia; chanly2009@cenat.gov.kh (C.N.); samoeurnkim@yahoo.com (S.K.); 7 National Health Commission, 1 Wainan Road, Xizhimen, Xicheng District, Beijing 100044, China; zhuge@nhfpc.gov.cn; 8 Chinese Center for Disease Control and Prevention, 155 Changbai Road, Changping District, Beijing 102206, China; zhanghui@chinacdc.cn (H.Z.); xuch@chinacdc.cn (C.X.); 9 Ministry of Health, Ban thatkhao, Sisattanack District, Rue Simeuang, 343 Vientiane, Lao PDR; sothbounmala@gmail.com (S.B.); senchanthixay@yahoo.com (M.S.); 10 National Center for Communicable Diseases Bayanzurkh district, Horoo 14, 13th district, Nam Yan Ju Street, Ulaanbaatar 210648, Mongolia; enkhtamiraaa@yahoo.com; 11 Ministry of Health, Government Building VIII, Olympic Street-2, Sukhbaatar District, Ulaanbaatar 210648, Mongolia; kh.ulzii@yahoo.com; 12 Health Policy Development and Planning Bureau, Department of Health, 2nd Floor Building 3, San Lazaro Compound, C. S. Gatmaitan Ave, Manila, 1003 Metro Manila, Philippines; paguiriganmd@gmail.com; 13 Department of Health-Region XI, J.P. Laurel Ave, Bajada, Davao City 8000, Philippines; hanselamoguis@gmail.com; 14 National Lung Hospital, 463 Hoang Hoa Tham Road, Ha Noi 124302, Viet Nam; xuanphu.vu@gmail.com; 15 Ministry of Labour-Invalids and Social Affairs, 12 Ngo Quyen Str, Hoan Kiem District, Ha Noi 110905, Viet Nam; khang.ortho@yahoo.com

**Keywords:** Tuberculosis, universal health coverage, social protection, Western Pacific Region, patient cost survey

## Abstract

Although the End TB Strategy highlights that major global progress towards universal health coverage (UHC) and social protection are fundamental to achieving the global targets for reductions in tuberculosis (TB) incidence and deaths, there is still a long way to go to achieve them in low- and middle-income countries. A workshop on the End TB Strategy Pillar 2 in the Western Pacific Region focusing on action towards UHC and social protection was held between 27 and 29 November in 2017 at the Korean Institute of Tuberculosis in Cheonju, Republic of Korea. The workshop brought together key personnel from national TB programmes and other stakeholders or researchers with experience in this topic from six countries with a high burden of TB in the region. During the workshop, participants shared country experiences, best practices, and challenges in achieving UHC and enhancing social protection in the context of TB service delivery, and also explored policy options to address the challenges, to be applied in their respective countries. This report describes the content of the meeting and the conclusions and recommendations arising from the meeting.

## 1. Introduction

Tuberculosis (TB) remains a major global health challenge, particularly among the poorest sections of society in low- and middle-income countries (LMICs). Although most countries aim to provide TB diagnosis and treatment free of charge within their health systems, many TB patients and their families are still suffering from financial hardship related to being ill with TB. A systematic review has shown that TB patients and their families in LMICs incur average costs amounting half of their household’s annual income due to TB [1]. Such a situation can be an obstacle to health care access and treatment adherence, thus causing increased risk of disease transmission and worse health outcomes. The End TB Strategy developed by the World Health Organization (WHO) acknowledges that poor access to health care and inadequate financial risk protection are major obstacles to ending the global TB epidemic [2]. The strategy highlights that major global progress towards universal health coverage (UHC) and social protection by 2025 are fundamental to achieving the global targets to reduce the TB incidence rate by 90% and the number of TB deaths by 95% by 2035 compared with 2015 [3]. In this respect, the strategy has set another target of “no TB affected families facing catastrophic costs due to TB” to be achieved by 2020 [3]. Further, the End TB Strategy has three pillars in which Pillar 2 of the strategy (i.e. bold policies and supportive systems) includes UHC and social protection as a major component, where the linkages to secure TB-sensitive UHC strategies should be developed and governments and stakeholders should ensure that TB is addressed in social protection and poverty alleviation schemes and programmes [3]. However, much remains to be done to reach UHC and social protection towards ending TB in LMICs. The Western Pacific Region, accounting for 17% of the global TB burden, also needs to move towards the full attainment of UHC and the expansion of social protection to end TB [4].

The Korean Institute of Tuberculosis (KIT) in collaboration with the WHO Regional Office for the Western Pacific held a workshop on the End TB Strategy Pillar 2 in the Western Pacific Region focusing on action towards UHC and social protection between 27 and 29 November in 2017 at the KIT in Cheonju, Republic of Korea. The workshop brought together key personnel from national TB programmes (NTPs) and other stakeholders or researchers with experience in this topic from six countries with a high burden of TB in the Region: Which are Cambodia, China, Lao People’s Democratic Republic (PDR), Mongolia, the Philippines, and Viet Nam. The workshop encompassed country progress reports, parallel group discussions, country action plans on UHC, and social protection as well as the results of TB patient cost surveys in some countries. The objectives of the meeting were to: Provide updates on UHC and social protection for TB care and prevention; share country experiences, best practices, and challenges in achieving UHC and enhancing social protection in the context of TB service delivery; and explore policy options to address the challenges and help measure baseline data and progress on UHC and social protection at the country level. This report describes the content of the meeting and the conclusions and recommendations arising from the meeting.

## 2. Country Progress on Universal Health Coverage and Social Protection for Tuberculosis Care and Prevention

The countries reported on the financing of TB services, the availability of health insurance relevant to TB services, drivers of TB patient costs, social support provided for TB patients, and successes and challenges in UHC and social protection.

As for the financing of TB services, TB diagnosis and treatment are supposedly free of charge in most of the participating countries. Although key TB services are included within national health insurance schemes in all countries except Cambodia, the percentage of population, service, and cost covered by health insurance is still insufficient. For instance, only 75% of TB patients in Viet Nam and 44% of medical costs in the Philippines are covered by national health insurance.

Most of the country participants regarded direct non-medical costs and income loss as a large proportion of patient costs, particularly after TB diagnosis. However, the description of how burdensome direct medical costs are for TB patients differed between countries depending on whether they had completed a TB patient cost survey or not (i.e., whether these had been directly measured). To illustrate, participants from Cambodia (where the survey has not yet been conducted) did not recognise direct medical costs after TB diagnosis as significant drivers of patient costs due to free TB services whereas participants from China (where the survey has been conducted) acknowledged that out of pocket (OOP) health expenditures after TB diagnosis were the main drivers of patient costs despite free of charge TB diagnosis and treatment (further details about the results of TB patient cost surveys are provided in Section 3).

The countries have various levels and types of support provided for TB patients. For example, Cambodia has a Health Equity Fund, which provides financial assistance to poor TB patients, Mongolia provides a disability allowance for TB patients, and Viet Nam has a living and vocational support programme for farmers with TB implemented by the Vietnam Famer’s Union. In addition, there are specific support programmes for multi-drug resistant TB (MDR-TB) patients in China, Mongolia, the Philippines, and Viet Nam in which hospitalisation, transportation, and/or food costs are subsidised.

There is overall progress in the coverage of health insurance and social protection for TB patients. However, the coverage still remains inadequate. In addition, many countries have a high level of dependence on external funding and poor access to health care and social protection schemes among marginalised populations in relation to challenges in UHC and social protection.

## 3. Patient Cost Surveys and Policy Implications

China, Mongolia, the Philippines, and Viet Nam have conducted their national TB patient cost surveys and are in different stages of results dissemination and policy dialogue. This report provides the average costs of a TB patient using three major cost categories and the percentage of TB-affected households facing catastrophic costs due to TB as well as the policy changes brought by the survey findings in the countries.

The main drivers of TB patient costs varied from country to country. Even within a country, the main drivers differed depending on the subgroup (Table 1). To be specific, direct non-medical costs and indirect costs were in the majority in the Philippines and Viet Nam. In Mongolia, the main drivers were direct medical costs among drug-susceptible TB (DS-TB) patients whereas they were direct non-medical costs among drug-resistant TB (DR-TB) patients.

**Figure 1:**
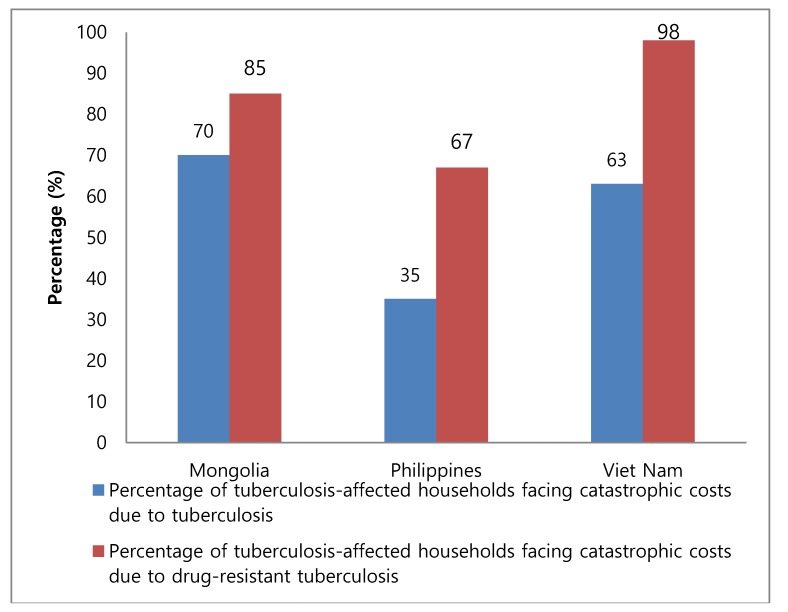
Percentage of tuberculosis-affected households facing catastrophic costs due to tuberculosis and drug-resistant tuberculosis in Mongolia, the Philippines, and Viet Nam.

The percentage of TB-affected households experiencing catastrophic costs was 70% in Mongolia, 35% in the Philippines, and 63% in Viet Nam (catastrophic costs due to TB are defined as total costs borne by patients exceeding a given threshold where 20% has been used as the threshold according to the current trend [5]). When limited to DR-TB patients, the percentage increased to 85% in Mongolia, 67% in the Philippines, and 98% in Viet Nam (Figure 1).

The survey findings have brought about policy changes in the countries. China proposed improving TB service delivery and intensifying health insurance schemes and social support to reduce the financial burden of TB patients. The Philippines proposed guidance on nutrition for TB patients to reduce nutritional supplementation costs, decentralisation of directly observed therapy (DOT) to reduce transportation costs, and application of a shorter regimen for DR-TB patients to reduce the proportion of those patients with catastrophic costs. There were many changes before and after the TB patient cost survey in Viet Nam. First, the Viet Nam NTP has launched a charitable foundation called the PAS TB foundation (Patient Support Foundation to End Tuberculosis), a funding mechanism for TB patients that can be used for purchasing health insurance for the poor and providing travel vouchers and food packages. Second, the NTP and the Ministry of Labour – Invalids and Social Affairs (MOLISA) are developing a roadmap to make existing general social protection schemes more TB-sensitive. Third, the NTP and the Ministry of Health (MoH) are developing a comprehensive package of ambulatory TB services. Fourth, the NTP, the MoH, and the MOLISA have started to collaborate on national policies on interventions to reduce TB patient costs. Fifth, the NTP is involving international partners to test new approaches to improve patient support and to reduce patient costs.

## 4. Interventions to Address Patient Catastrophic Costs

The participants were divided into two groups (at least one participant for one group from each country) for parallel discussions where one group (group 1) focused on interventions to address direct medical costs and the other one (groups 2) focused on interventions to address direct non-medical costs and income loss.

Group 1 proposed improving the patient pathway to TB diagnosis and treatment as interventions to address direct medical costs. To be specific, improving awareness, expanding coverage of new technologies and services even in the private sector, decentralisation of health care services, expansion of active case finding (ACF), reducing hospital stay, and standardisation of treatment protocols were suggested options. In addition, the group proposed health insurance reforms, such as improvements in health insurance coverage, reducing co-payments, and capping of the level of payments for OOP health expenditures.

Group 2 proposed conditional cash transfers, the provision of food and transportation, expansion of community-based care and collaboration of health and social services as interventions to address direct non-medical costs as well as broader poverty reduction efforts, legislation to protect workers, and sickness and unemployment benefits as interventions to address income loss.

## 5. Country Actions towards Universal Health Coverage and Social Protection for Tuberculosis Care and Prevention

Each participating country presented a summary of country actions towards UHC and social protection. The presentations included the current status of TB patient cost survey and actions to reduce direct medical costs, direct non-medical costs, and indirect costs.

With regard to the current status of national TB patient cost surveys, Viet Nam had disseminated the results and progressed to policy dialogue and action planning based on the survey findings, China and the Philippines had just completed the analysis, and Mongolia was in the process of finalising the analysis. Other countries were preparing their surveys.

Most of the countries proposed not only enhancing TB service delivery, but also improving TB care financing to reduce direct medical costs. To enhance TB service delivery, Mongolia is expanding outpatient clinics to provide TB ambulatory services and the Philippines have been establishing a Service Delivery Network (SDN) wherein health care providers are organised to ensure easy access to high quality services, such as chest X-rays, across health facilities. To improve TB care financing, China is reforming the payment of health insurance, thus reducing the co-payment portion of TB patients, and Viet Nam has launched a charity fund to purchase health insurance for poor TB patients.

Most of the countries proposed not merely TB-specific interventions, but also improved linkages with general social protection schemes to reduce direct non-medical costs and indirect costs. For TB-specific interventions, China, Mongolia, the Philippines, and Viet Nam have specific support programmes for MDR-TB patients in order to subsidise hospitalisation, transportation, and/or food costs. To improve access to social protection schemes, Mongolia has involved local employment offices and relevant sectors to provide employment support services to unemployed TB patients and the Viet Nam NTP and MOLISA are developing a roadmap to make existing general social protection schemes more TB-sensitive.

## 6. Conclusions and Recommendations

The workshop was the first to specifically deal with UHC and social protection for TB care and prevention in the region. During the workshop, participants shared country experiences, best practices, and challenges in achieving UHC and enhancing social protection in the context of TB service delivery, and also explored policy options to address the challenges, to be applied in their respective countries.

The findings and policy implications of the national TB patient cost surveys from four countries inspired other country participants. It was found that the main drivers of TB patient costs varied between and within countries. In addition, we observed that national action planning was influenced by the findings of the national TB patient cost surveys. For example, China is focusing on intensifying primary health care and reforming health insurance to reduce direct medical costs while Viet Nam is focusing on expanding TB-specific interventions and enhancing social protection schemes to reduce direct non-medical costs and indirect costs.

Participants explored policy options to alleviate financial hardship for TB patients. Patient costs can be mitigated by enhancing health service delivery towards patient-centred care, such as decentralised services, and by improving TB care financing in line with progress towards UHC. In addition, the remaining costs even after health service enhancement can be reduced by intensified social protection schemes.

Based on full consultation, we recommend the following actions towards UHC and social protection for TB care and prevention in countries with a high burden of TB in the region.

First, national TB patient cost surveys should be conducted. These surveys help understand which cost categories are the main drivers of TB patient costs in order to prioritise policies to mitigate the financial burden of TB patients. In addition, these surveys are used to determine the baseline and periodically measure the percentage of TB-affected households facing catastrophic costs due to TB in line with the End TB Strategy.

Second, interventions to alleviate financial hardship for TB patients primarily under the responsibility of the NTP or MoH should be implemented immediately. Policy options, such as decentralised DOT, expansion of ACF, and increased health insurance coverage for TB-related services, can be quickly realised with strong political commitment.

Third, specific interventions for MDR-TB patients should be intensified. Several national TB cost surveys have demonstrated that a much higher proportion of MDR-TB patients face catastrophic costs compared with DS-TB patients. Hence, general measures to mitigate the financial burden of TB patients may be insufficient for MDR-TB patients and further actions may need to be taken to reduce the proportion of those patients facing catastrophic costs.

Fourth, multi-sectoral stakeholders should be engaged in order to provide improved forms of social support for TB patients. Many social interventions for TB patients require collaboration with stakeholders beyond the health sector. If all key stakeholders take concrete actions to alleviate financial hardship for TB patients, we will be able to achieve significant progress towards social protection for TB patients.

Lastly, a research platform should be established and operationalised. Research can be utilised not only to understand the main barriers and opportunities to progress towards UHC and social protection, but also to design and evaluate the impact of UHC and social protection.

## Figures and Tables

**Table 1:** Tuberculosis patient costs in Mongolia, the Philippines, and Viet Nam (USD).

Cost Category	Mongolia		Philippines ^1^			Viet Nam	
	DS-TB	DR-TB	Urban DS-TB	Rural DS-TB	DR-TB	DS-TB	DR-TB
Medical costs	215 (43.0%)	438 (33.0%)	9.6%	6.8%	2.1%	134 (12.6%)	784 (18.3%)
Non-medical costs	195 (39.0%)	598 (45.0%)	26.7%	52.1%	62.7%	414 (38.8%)	2141 (49.9%)
Income loss	90 (18.0%)	292 (22.0%)	63.7%	41.1%	35.2%	519 (48.6%)	1363 (31.8%)
Total costs	500 (100.0%)	1328 (100.0%)	100.0%	100.0%	100.0%	1067 (100.0%)	4288 (100.0%)

TB: tuberculosis; DS-TB: drug-susceptible TB; DR-TB: drug-resistant TB; ^1^ No data were available except percentage of cost category among TB-affected households facing catastrophic costs.

## References

[1] Tanimura T., Jaramillo E., Weil D., Raviglione M., Lonnroth K. (2014). Financial burden for tuberculosis patients in low- and middle-income countries: A systematic review. Eur. Respir. J..

[2] Lonnroth K., Glaziou P., Weil D., Floyd K., Uplekar M., Raviglione M. (2014). Beyond UHC: monitoring health and social protection coverage in the context of tuberculosis care and prevention. PLoS Med..

[3] World Health Organization (2015). Implementing the End TB Strategy: The Essentials.

[4] Rahevar K., Fujiwara P.I., Ahmadova S., Morishita F., Reichman L.B. (2018). Implementing the End TB Strategy in the Western Pacific Region: Translating vision into reality. Respirology.

[5] World Health Organization (2017). Tuberculosis Patient Cost Surveys: A Handbook.

